# Viral Myositis in a Pediatric Patient Following Influenza Infection: A Case Report

**DOI:** 10.7759/cureus.71294

**Published:** 2024-10-12

**Authors:** Joshua A Jogie, Ayaan Arain, Jeremy Jogie, Travis Satnarine, Shaista Arain

**Affiliations:** 1 Faculty of Medical Sciences, The University of the West Indies, St. Augustine, TTO; 2 Department of Physiology, University of Kentucky, Lexington, USA; 3 Family Medicine, Chaguanas District Health Facility, Chaguanas, TTO; 4 Pediatrics, University of Miami Miller School of Medicine, Jackson Memorial Hospital, Florida, USA; 5 Department of Pediatrics, University of Kentucky Children's Hospital, Lexington, USA

**Keywords:** creatine kinase elevation, influenza a, pediatric infection, supportive care, viral myositis

## Abstract

Viral myositis is an uncommon sequela associated with influenza virus infection, more common in children. We report a case of a three-year-old girl who developed viral myositis after suffering from influenza A infection. The patient was found to have leg pain associated with difficulty in walking. The diagnosis was confirmed by physical examination and elevated creatine kinase (CK) level. Supportive measures, including rehydration and mitigation of fever, were sufficient. Antiviral therapy was not required. This case also underscores the importance of early diagnosis and supportive medical care in managing children with viral myositis. Persistently elevated levels of CK are also useful in diagnosis as they provide evidence of muscle damage sustained during virus infection. The immediate concern would be the reassurance of caregivers regarding cases that will almost always be resolved with supportive care instead of worsening progressively. Prevention of unnecessary courses of management will decrease the patient's anxiety and the parents' fears.

## Introduction

Viral myositis is rare but is a known complication of influenza infections in children. It encompasses the inflammation and weakness of muscles and often results in difficulty walking, as depicted in the presented case of a three-year-old girl with influenza A. Symptoms include cold or flu-like disease, muscle pain, and difficulty walking, after which these patients recover in only a few weeks with appropriate management [1]. Early diagnosis and sufficient measures are crucial to avoid further complications and ensure full recovery.

Myositis is frequently a complication of influenza virus infections in children. It occurs most commonly following an influenza A viral infection. This condition is associated with pain or tenderness in calves while walking, unwillingness to walk, and a self-limited course. Despite its frequency, medical doctors must recognize viral myositis and differentiate it from other causes of muscular weakness. This will allow them to inform caregivers of its benign prognosis and reassure them [2,3]. In this instance, the patient was treated with supportive therapy, including fluids, antipyretic treatment, and watchful waiting.

Laboratory findings demonstrating an increase in the levels of creatine kinase (CK) and, in some cases, positive influenza test results can support clinical findings and confirm viral myositis [4]. Such clinically significant muscle damage leading to increased serum CK levels may indicate active myositis in patients [5]. Symptoms of flu infection with muscular pain should alert health professionals of the possibility of viral myositis, prompting proper intervention [3]. This case underlines the risk of complications of influenza infection and the importance of early detection and timely management, as these can significantly affect the progression of the disease in the patient [6,7].

## Case presentation

A three-year-old female reported a runny nose, cough, and congestion. These symptoms started three days ago. She also had a fever for the first three days with temperatures as high as 102 °F, which was controlled with four to six hourly doses of acetaminophen and ibuprofen. The fever had resolved by the time of the clinic appointment. The child’s fluid intake and appetite were appropriate, and there was no vomiting or diarrhea. She had been urinating and passing stool without any problems. The child’s sister was also confirmed to have influenza three days before this appointment.

On the day before the visit, the parents reported that she had leg pain and refused to walk, which persisted during the visit. This same complaint persisted on the day of the visit. They were worried that she could not walk or move much in general and how uncomfortable she looked. Developmental and neonatal histories were unremarkable. The child is up-to-date with all vaccinations. Clinically, the patient was well without respiratory distress, and her vitals were stable. She was rather reluctant to move and walked only when specifically requested, with an unsteady gait. Tip-toe walking was noted, with some avoidance of putting the entire foot on the ground. However, on physical assessment, there was a normal range of motion (ROM) in her extremities and no tenderness of the calves and thighs.

The first laboratory investigations reported were a positive polymerase chain reaction (PCR) for influenza A and a considerable leukocyte elevation to 15.22 thou/µL, with neutrophil dominance at 74.4%. There were significant effects of muscle breakdown, as evidenced by an elevated level of CK at 673 U/L. The rest of the laboratory tests, such as comprehensive metabolic panel (CMP) and urine examination, were generally unremarkable. There was no growth from blood and urine cultures after two days.

The child was diagnosed with influenza A-associated myositis. Supportive measures were resumed, including antipyretics and hydration maintenance. The antiviral medications (oseltamivir) were not taken because the parents declined. After two days, the next visit showed improvements in the child's gait, albeit she had a temperature as high as 102.5 °F. During this follow-up, she could walk steadily without complaining of discomfort or leg pain. In the days following this, the patient remained afebrile. A full recovery was expected with continued supportive management.

## Discussion

Viral myositis is a rare but well-documented sequela of influenza infection, especially in children. Our case illustrates the classical triad presentation of this disease: sudden onset muscle pain and impairment of ambulation in a child previously diagnosed with influenza A. Research confirms that viral myositis often occurs after the acute influenza infection phase [1,5]. In this case, the child suffered from leg pain and gait abnormalities, which correlated with viral myositis features. This disease is self-limiting and responds to supportive treatment [8].

Moreover, CK elevation is a pertinent diagnostic criterion for viral myositis, as demonstrated in this case report. Increased levels of CK indicate the presence of muscle damage, usually seen in association with viral myositis following the influenza virus [9]. Studies have shown that the extent of CK rise is sometimes proportional to the extent of muscle symptoms presented, but levels normalize as the condition resolves [10]. In this patient, the CK value of 673 U/L verified the diagnosis and was in line with other case reports of influenza-associated myositis [11].

The management of viral myositis is mainly supportive, with hydration and antipyretics being the key interventions. Antiviral treatment is not mandatory for recovery in viral myositis, as this case illustrates when the parents refused antiviral therapy. This is consistent with the existing literature suggesting that most cases of viral myositis in children are self-limiting and last about one to two weeks [2]. This is further evidenced by the child’s improvement during follow-up, which resolved without specific antiviral therapy [3].

Other causes of acute muscle weakness and pain include, but are not restricted to, Guillain-Barré syndrome, bacterial myositis, and others, which all need to be ruled out when diagnosing viral myositis [12]. Despite the differential diagnosis, the clinical and laboratory presentation of the patient, including elevated CK levels and PCR positivity, confirmed viral myositis. It is very important to recognize these conditions as early as possible to avoid unnecessary invasive tests or treatments because of misdiagnosis [13].

## Conclusions

Viral myositis in children is a rare illness requiring early recognition. In this case, however, supportive measures, such as hydration and antipyretics, were effective. Antiviral treatment was not needed to bring about the patient’s recovery. Elevated creatine kinase levels associated with the diagnosis were noteworthy and further supported findings in similar studies. The gradual improvement in the patient over a few days suggests that, in many instances, this diagnosis is self-limiting. Differentiation of viral myositis from other causes of muscle weakness is important. This will prevent unwarranted treatments and provide reassurance to both caregivers and patients.
